# Neutron imaging of an operational dilution refrigerator

**DOI:** 10.1038/s41598-022-05025-0

**Published:** 2022-01-21

**Authors:** C. R. Lawson, A. T. Jones, W. Kockelmann, S. J. Horney, O. Kirichek

**Affiliations:** ISIS Neutron & Muon Source, Rutherford Appleton Laboratory, Chilton, Didcot, OX11 0QX Oxfordshire UK

**Keywords:** Quantum fluids and solids, Imaging techniques

## Abstract

The invention of the ^3^He/^4^He dilution refrigerator opened a new chapter in experimental ultra-low temperature physics. Dilution refrigerators became essential for providing ultra-low temperature environments for nuclear demagnetisation experiments, superconducting-qubit quantum processors and highly sensitive bolometers used in fundamental physics experiments. Development of dilution refrigeration technology requires thorough understanding of the quantum mechanical processes that take place in liquid helium at ultra-low temperatures. For decades the quantum fluids research community provided valuable information to engineers and designers involved in the development of advanced dilution refrigerators. However, the lack of methods that allow the measurement of physical parameters of liquid helium during the operation of a dilution refrigerator was hindering development of the technology. Here we show direct imaging of an operational dilution refrigerator using neutron radiography. This allows direct observation of the dilution process in ^3^He/^4^He mixtures and opens an opportunity for direct measurement of the ^3^He concentration. We observe the refrigerator behaviour in different regimes, such as continuous circulation and single shot, and show that our method allows investigation of various failure modes. Our results demonstrate that neutron imaging applied to the study of dilution refrigeration processes can provide essential information for developers of ultra-low temperature systems. We expect that neutron imaging will become instrumental in the research and development of advanced dilution refrigerators.

## Introduction

Today, dilution refrigeration is the most widespread technology for accessing the temperature range between about 5 mK and 1 K, and is the only technique capable of continuous operation in this temperature range. It is capable of producing large cooling powers with relatively modest pumping arrangements by making use of the enthalpy of mixing when ^3^He atoms move across a boundary from a phase of concentrated ^3^He to a phase of dilute ^3^He^1^. The dilution refrigerator (DR) is quite simple in construction, easy to operate, and can work in high magnetic fields (as is often required in ultra-low temperature experiments)^2^. It is also used in sub-millikelvin systems as a base from which lower temperatures can be reached. The idea of using the entropy of mixing of ^3^He and ^4^He isotopes for refrigeration was first suggested in 1951 by London^3^, followed a decade later by the proposal of continuous refrigeration for temperatures below 300 mK, published by London et al.^4^.

The first working prototype of a refrigerator based on this principle was built by Das et al. in Leiden University in 1965, reaching a temperature^5^ of 0.22 K; the lowest temperature obtained in helium liquids at that time. Only 1 year later, Neganov et al. at JINR, Dubna^6^, and Hall et al. at the University of Manchester^7^ published results obtained on their prototype ^3^He/^4^He DR based on improved designs. In both cases they managed to reduce the base temperature significantly below 100 mK. A comprehensive review of the dilution refrigeration method and technology has been published by Wheatley et al.^8^. The lowest DR base temperature to date is ∼1.75 mK and it has been achieved in an advanced DR designed for the Lancaster Microkelvin Facility^9^. The DR was operated in continuous mode and could run at the base temperature for 10 days between refrigerant refills.

The introduction of closed cycle refrigerators (CCRs) to DRs in the 1990s was a turning point in modern DR development. With a sufficiently cold CCR, one is able to substitute the ^4^He bath of a DR cryostat with a closed cycle device. The obvious advantage of this approach is the removal of liquid ^4^He, which is both awkward to handle and increasingly expensive. Indeed, the current prices of liquid ^4^He (~ £15/l) most likely prohibits many institutions, who may not have helium recovery infrastructure, from using ‘wet’ cryogenics. Instead, one can run a CCR from an electrical socket. Initial attempts to integrate a CCR into a DR used a Gifford–McMahon (GM) style device, however modern systems will use a pulse tube refrigerator (PTR) to take advantage of the lower temperature and smaller level of vibrations offered by a PTR. A comprehensive review describing the growth of cryogen free DRs can be found in reference^10^, with an example specific to neutron scattering available in reference^11^. Other developments which have occurred, thanks to the freedom designers have when not limited by the requirement for a liquid ^4^He dewar, include increasingly large mixing chambers (currently around 1 m), more open experimental space, the use of a single vacuum space for the whole DR, use of multiple DR units in one system, and the evolution of computer control systems.

Beyond this, the technical development of DRs has evolved into two basic directions. The most intensive engineering efforts were dedicated to increasing cooling power, simultaneously with maintaining the lowest base temperatures. High power DRs can be found in applications such as: sub-millikelvin nuclear demagnetisation experiments^9^, cooling of superconducting-qubit quantum processors^12–14^ and fundamental physics experiments that require ultra-low temperatures for highly sensitive bolometers like the cryogenic underground observatory for rare events (CUORE)^15^, or extremely low noise displacement sensors used in the MiniGRAIL resonant mass antenna for detection of gravitational waves^16,17^.

Another direction of DR development concentrated on minimising the time required to cool samples from room temperature to the base temperature of the DR. This approach allows quick characterisation of large numbers of samples as is often required in the areas of low temperature magnetism, superconductivity and qubit testing. Such DRs are also widely used for neutron scattering and muon spectroscopy sample environments^18^, and many of the DRs available at neutron and muon facilities are based on the Grenoble design that incorporates sintered silver heat exchangers^19,20^. Today a variety of ‘quick’ DRs are offered by a number of commercial companies.

Here we describe the application of neutron imaging techniques to an operational dilution refrigerator, which allows direct observation of the dilution process in ^3^He/^4^He mixtures, and provides an opportunity to directly measure important parameters of the liquid helium mixture. We studied the DR in different operational regimes, where the well-known behaviours were strikingly revealed by neutron imaging. We also simulate a common failure mode of DRs, where there is insufficient ^3^He in the mixture. The preliminary images from this experiment show the potential power of the technique in the design and understanding of advanced, or unusual, DRs.

## Experimental setup

The measurements were performed on the IMAT instrument at the ISIS neutron and muon source, which provides neutron radiography and tomography, and energy-resolved neutron imaging^21^. Neutrons are well suited for transmission through the metal components of the sample environment, such as a cryostat or DR, but at the same time are very sensitive to the ^3^He isotope that allows us to follow the diffusion of ^3^He in a ^3^He/^4^He mixture. Basic specifications of the instrument together with settings used in our experiment can be found in the “Methods” section.

**Figure 1:**
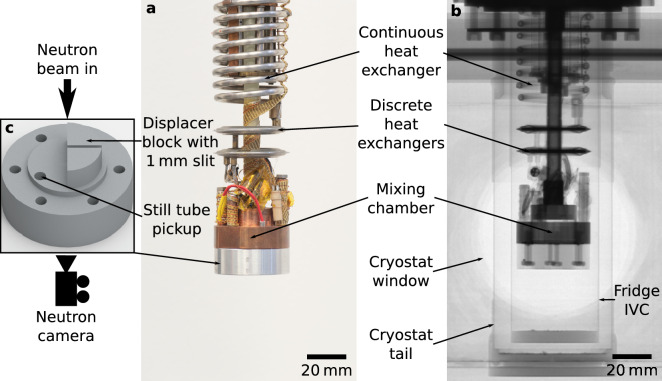
The dilution refrigerator used for this study. (**a**) The dilution insert as viewed under visible light, and (**b**) shows it, to the same scale, under neutron imaging using the IMAT instrument. (**c**) The base of the mixing chamber used for this investigation. This features a displacer block with a 1 mm gap to allow imaging of the helium mixture without the excessive absorption associated with neutrons passing through mixture over the full 22 mm-diameter mixing chamber. The incident neutron beam is perpendicular to the plane containing the slit, and parallel to the straight edge of the displacer block, as shown by the large black arrow.

In our experiment we used a KelvinoxJT^®^ DR produced by Oxford Instruments Nanoscience, designed for delivering an ultra-low temperature sample environment for neutron scattering experiments in the range 30 mK–2 K. Neutron and optical images of the same empty DR unit are presented in Fig. 1. The neutron image has been taken with the DR insert loaded into the cryostat, and therefore includes the thermal shield and vacuum cans, however these are almost transparent for the neutron beam. Major components of the DR unit, such as the mixing chamber (MC), two discrete heat exchangers, continuous heat exchanger and interconnecting tubing, are clearly recognisable in the neutron image. The technical specification of the standard KelvinoxJT^®^ and details of the cryogenic set up can be found in the “Methods” section.

Neutrons are extremely sensitive to ^3^He, which has an absorption cross section of 5333 b for 1.8 Å neutrons, whereas ^4^He is almost transparent^22^. This vast difference in absorption cross section allows neutron imaging to clearly distinguish between ^3^He and ^4^He. This effect has been used previously in neutron radiograph experiments performed at the ILL, which showed ^3^He droplets forming in ^4^He inside the return capillary of a dilution refrigerator designed for space applications^23^. However, attempts to perform similar imaging of a traditionally designed dilution refrigerator’s MC encounter a major obstacle, because even a relatively small amount of ^3^He (e.g. 6.6% in the weak phase of the ^3^He/^4^He mixture) efficiently adsorbs almost all neutrons, leading to poor contrast between the weak and concentrated phases in the MC. In order to resolve this problem, we modified the MC such that one half of it can sustain a reduced circulation of ^3^He in the DR (for continuous operation), leaving another half for the imaging of ^3^He/^4^He mixture phase separation. Most of the imaging half of the MC is occupied by an aluminium displacer which is relatively transparent to neutrons. In the middle of the displacer there is a 1 mm gap into which mixture can enter. A CAD model of the MC displacer is shown in Fig. 1c. Taking the spectrum-weighted average neutron wavelength used on IMAT, one can use the Beer-Lambert law to calculate the neutron transmission through 1 mm of pure ^3^He and through 6.6% ^3^He/^4^He mixture as 10^–6^% and 12.5%, respectively. This arrangement allows us to observe the phase separation of the helium mixture in the gap below 0.8 K.

## Experimental results

**Figure 2:**
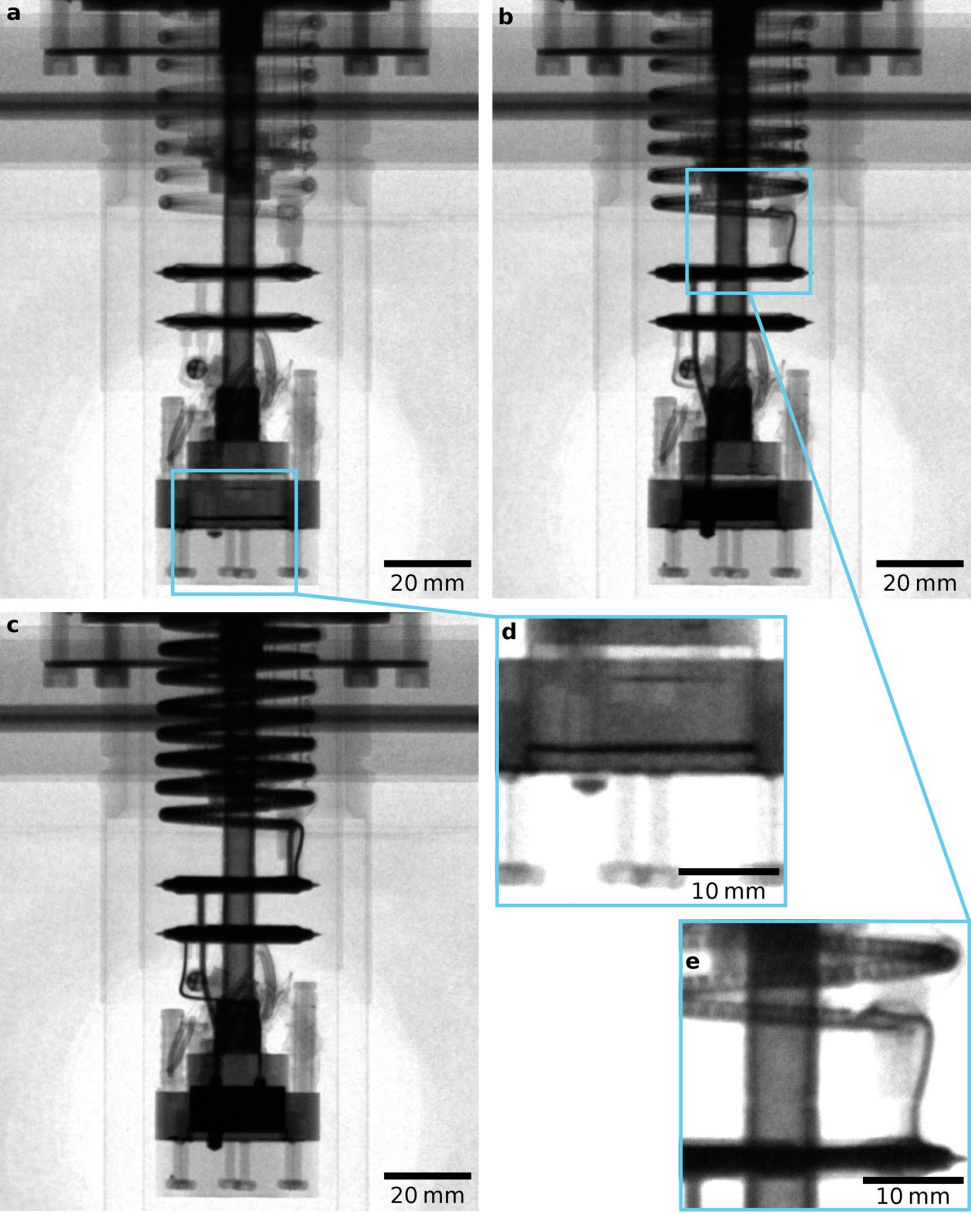
Neutron images of the DR during mixture condensation. (**a**–**c**) The DR progressing through the condensation process. Note the small layers of mixture in the still tube pickup, at the MC base, and on top of the displacer in the inset (**d**), which is a zoomed-in and contrast-adjusted region of (**a**), captured shortly after the initiation of condensation. Panel (**e**) is a zoomed-in and contrast-adjusted region of (**b**) showing mixture flowing through the inner tube of the continuous heat exchanger. A video of the full condensation process is in Supplementary Video S1.

We study the DR’s behaviour when operated in different modes, including initial helium condensation, phase separation of the ^3^He/^4^He mixture, continuous circulation, and the single shot regime. The process of initial condensation is shown in Supplementary Video S1, where the turbulent and oscillatory nature of the process is clearly displayed. Due to surface tension and wetting effects^24^, the first stable liquid forms on the top of aluminium displacer *as well as* in the bottom of the MC, visible as a thin layer in Fig. 2d. Approximately 45 min after starting the condensation process, the liquid helium fills the observable part of the DR unit with no sign of boiling or other disturbances, see Fig. 2c.

### DR in continuous circulation

Once the condensation procedure is complete and the mixture has phase-separated in the MC, the DR moves into a regime of continuous circulation. In this regime the DR exists in a state of dynamic equilibrium, where almost pure ^3^He is removed from the still by distillation and reinjected through the condensing circuit. This results in ^3^He atoms moving continuously across the phase boundary in the MC from the concentrated to the dilute phase.

**Figure 3:**
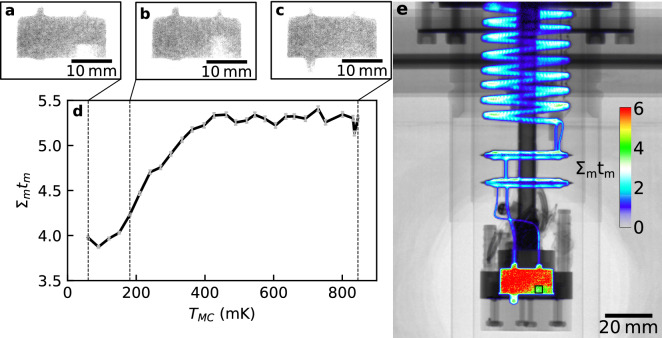
Changing ^3^He concentration with temperature in the mixing chamber of the DR. Panels (**a**–**c**) show neutron images of the mixing chamber of the DR at the three temperatures indicated in (**d**), which is a plot of the quantity Σ_m_t_m_ against mixing chamber temperature. Note how Σ_m_t_m_ increases with temperature, indicating further attenuation of the neutron beam and hence greater ^3^He concentration in the dilute phase; and the phase boundary moves upwards, as expected because of the resulting redistribution of the mixture. The standard deviation of each data point in (**d**) is shown by the vertical error bars. Panel (**e**) shows the quantity Σ_m_t_m_ throughout the entire dilution refrigerator circuit for a mixing chamber temperature of 60 mK and indicates the measurement position for the graph in (**d**) with the black square at the lower-right of the mixing chamber.

In order to study the continuous operation of the DR, we imaged the DR as a function of temperature. The MC temperature was slowly increased in 30 mK increments from 60 to 850 mK, as determined using a Lakeshore 370 AC resistance bridge measuring a calibrated RuO_2_ sensor that was thermally anchored to the MC wall. At each temperature step the DR was allowed to settle, before being imaged over 8 min (see “Methods”). The captured images (Fig. 3a–c) show the neutron transmission through the dilution refrigerator and helium mixture. This data was then processed to calculate the quantity Σ_m_t_m_ (Fig. 3d,e), where Σ_m_ is the macroscopic cross section of the helium mixture and t_m_ the neutron path length through it. The quantity Σ_m_ is directly related to the concentration of ^3^He in the mixture, see “Methods”.

### DR single shot

To ‘single shot’ a DR is the procedure by which re-introduction of ^3^He into the condensing circuit is halted, the only process then remaining is the movement of the remaining ^3^He from the concentrated to dilute phase, before it is removed from the system at the still. This will continue until all ^3^He is removed from the system, with only ^4^He remaining (due to its vanishingly small vapour pressure at still temperatures, practically no ^4^He will be extracted during the single shot procedure). This means there is no longer a heat load originating from the comparatively warm ^3^He re-entering the MC. The single shot procedure serves as a diagnostic of DR issues, most notably the accurate determination of the mixture’s ^3^He to ^4^He ratio.

**Figure 4:**
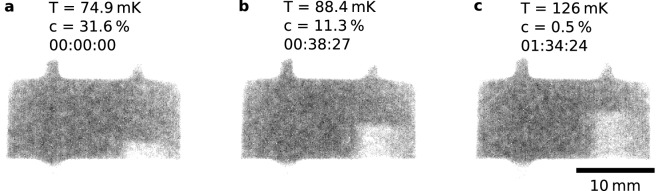
Mixing chamber of the DR during a single-shot operation. Panel (**a**) shows the initial position of the phase boundary before starting to remove the ^3^He from circulation, panel (**b**) shows the condition when the ^3^He concentration has dropped to 11%, and panel (**c**) is with almost all the ^3^He removed. A video of the single-shot process is in Supplementary Video S2. In all three panels, an annotation of the mixing chamber temperature (T), the ^3^He concentration (**c**) and the time since beginning the single shot (HH:MM:SS) is given.

The single shot procedure was performed until virtually all ^3^He was returned from the DR to the gas handling system. Throughout the single shot, neutron imaging measurements were made (see “Methods”). As with the continuous mode result, one would expect the phase boundary between concentrated and dilute phases to move up the MC as ^3^He is removed from the liquid state, visible in Fig. 4.

### Effect of incorrect mixture

Although the imaging of the expected DR operations, procedures and behaviours is both informative and fascinating, the real power of the neutron imaging technique demonstrated in this paper is the potential to investigate unusual DR performance or malfunctions. One of the more common failure modes for a DR is the use of incorrect mixture quantity, or ^3^He concentration. It is therefore interesting to image the DR in a situation where the concentration of ^3^He in the mixture is below the optimum value.

**Figure 5:**
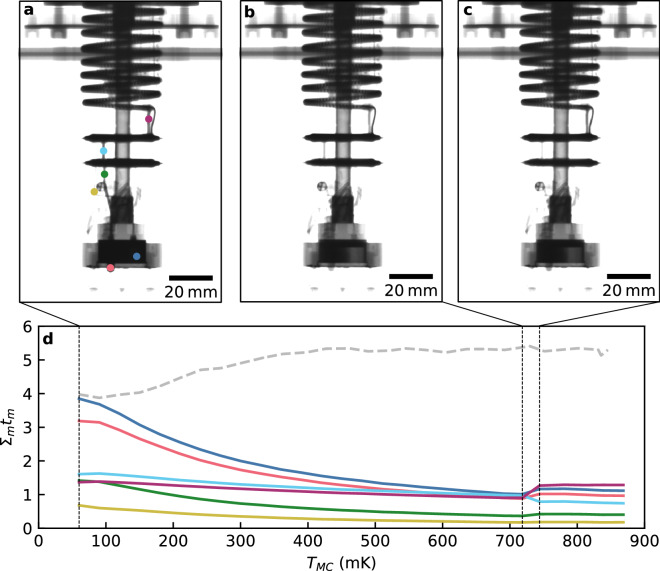
Operation of the dilution refrigerator with insufficient ^3^He (28%). Panels (**a**–**c**) show neutron images of the operating DR, contrast adjusted in order to more clearly show the mixture. Panel (**d**) shows the quantity Σ_m_t_m_ for neutrons penetrating several parts of the DR, as indicated by the coloured circles in panel (**a**). Note that the neutron absorption in the mixing chamber initially reduces with increasing temperature, the opposite of what would normally be expected (see the dashed grey line, reproduced from the Fig. 3 data), and then changes abruptly around 750 mK.

This experiment was performed in an identical way as that of normal continuous operation, but with insufficient ^3^He in the mixture (28% instead of 32%). The results, visible in Fig. 5, show unusual behaviour. Most notably the neutron absorption in the mixing chamber (and therefore the concentration of ^3^He) initially reduces with rising temperature, the opposite of what would normally be expected, and then changes suddenly around 750 mK. Additionally, we observed no visible phase separation in the imaged section of the MC (see Fig. 5a–c, where only the top of the displacer is visible), which suggests that phase separation may have occurred outside the MC. A modified setup may allow us to observe the exact location of the phase boundary and directly read-out the mixture concentrations.

## Discussion

The images and data captured during normal DR operation, and the single shot procedure, give an insight into the real movement of helium within the dilution circuit. Analysis of the images allows us to give an indication of how the ^3^He concentration in the mixture changes with both time and temperature.

The temperature of the MC itself is dictated by the balance of the thermal load versus the cooling power of the DR. The maximum possible cooling power in W, $\dot{Q}$, of the DR in this mode is given by^2^

1 $$\dot{Q}=84 {\dot{n}}_{3}{T}^{2},$$

where T is the temperature of the MC in K and ${\dot{n}}_{3}$ is the amount of ^3^He transferred from the concentrated to the dilute phase per unit time, in mol/s (referred to as the circulation rate). The cooling power can be increased by warming the still, since this leads to a greater saturated vapour pressure of ^3^He above the still, and hence a greater circulation rate. The still temperature is usually controlled using an electrical heater mounted to the still itself, however it is also affected by the heat load from the circulating helium, leading to the situation where elevated heat loads (and therefore temperatures) lead to more ^3^He in the gas phase.

For T → 0, the concentrated phase at the top of the mixing chamber consists entirely of pure ^3^He and the dilute phase has 6.6% ^3^He^25^. As the temperature is increased, the ^3^He concentration in the concentrated phase decreases, and in the dilute phase increases, until the boundary of the phase separated region is reached (at 870 mK for an average overall concentration of 67.5%, lower for other concentrations).

The result of these two effects is that higher temperatures alter the ratio of ^3^He–^4^He in the MC. Since the overall mixture concentration is fixed, this variation in concentrations necessarily leads to the relative quantities of each phase varying. In this case, where the overall concentration is 32% (less than the tricritical concentration of 67.5%), the quantity of dilute phase will increase due to the ^3^He concentration increasing with temperature, i.e. the phase boundary will move upwards on warming the MC. At temperatures above the miscibility gap, phase separation will be lost and the dilution process will no longer be in operation.

The results from our study of the continuous circulation regime are consistent with this well-established theory of DR operation. However, the neutron imaging technique applied to operation with insufficient ^3^He revealed two unusual effects. Firstly, the phase separation boundary appears to form outside of the imaged region (and therefore most likely outside the MC) and secondly, the concentration of ^3^He in the MC reduces with increasing temperature.

Whilst further investigation (involving higher resolution measurements of a much larger fraction of the DR mixture circuit) would be needed for a complete description of the phenomena, these initial measurements show how powerful the technique would be in the understanding of unusual DR systems, such as those in^24, 25^ or those used for space applications. In particular, the ability to directly read out the ^3^He concentration would be extremely useful, and could be tested in a future study by using a monochromatic neutron beam to avoid beam-hardening effects (see “Methods”), itself enabled by a thinner, two dimensional mixing chamber with less shielding in order to reduce the overall beam attenuation.

## Conclusion

The dilution refrigerator is a ubiquitous piece of equipment, used in practically all experimental condensed matter laboratories. It has developed over the course of half a century into a modern, mass-produced provider of millikelvin temperatures. Coupling this low temperature workhorse with the powerful advanced neutron radiography instruments available today, we have been able to demonstrate in-situ imaging of the dilution process, giving an insight into effects that, whilst long understood and well measured, had not yet been truly seen.

Direct measurement of the DR’s condensing, circulation, and single shot modes has provided a rich set of images and videos which give a clear picture as to the processes hidden within. We believe this is an invaluable resource for students, educators and professionals alike. The application of this technique to the area of fault-finding and investigation, as exhibited in this paper, opens up a more direct and thorough route through which designers and engineers can continue to push the limits of this low temperature technology.

## Methods

### Neutron imaging

IMAT (Imaging and Materials Science & Engineering)^21^ is a neutron imaging and diffraction instrument that currently provides neutron radiography, neutron tomography and energy-resolved neutron imaging. IMAT is installed on the second target station at the ISIS pulsed neutron source, United Kingdom. The source operates at 10 Hz and provides a liquid H_2_ moderator at < 20 K. A neutron wavelength range between 1 and 6.7 Å was selected, for a spectrum that peaks at about 2.6 Å and with an average wavelength of about 3.3 Å. The experiment used a ‘pinhole setup’, with an aperture of diameter D at a distance of L = 10.2 m to the mixing chamber. For most of the images reported in this paper, a pinhole D = 20 mm (aspect ratio L/D ~ 500) was used. The neutron flux^21^ was about 1.5 × 10^6^ n/cm^2^/s. Some test scans and the scans shown in Fig. 4 were performed with D = 40 (L/D ~ 250).

The neutron camera used a ZnS/LiF:Cu scintillator of 200 µm thickness and an ANDOR sCMOS Zyla 4.2 module with 2048 × 2048 pixels (Oxford Instruments, UK). The sCMOS viewed the scintillator screen via a lens with focal length of 50 mm and numerical aperture of f = 1.2 via a 45° mirror, providing a pixel size of 0.103 mm and a field of view of 211 × 211 mm^2^. A neutron beam size of square cross section 200 × 200 mm^2^ was selected using a set of neutron-absorbing beam limiters between the pinhole and cryostat. The effective spatial resolution of the neutron imaging setup was 0.53 mm, dominated by the geometric blur which was given by the distance of 270 mm of the mixing chamber from the scintillator screen, divided by L/D.

The dilution refrigerator was mounted on a positioning table in front of the neutron camera, and as close as possible to the neutron camera screen. At the start of the experiment several dark images (with the neutron source off) and ‘open beam’ images (with the cryostat removed) were collected.

### Dilution refrigerator

For our experiment we used an Oxford Instruments KelvinoxJT^®^ dilution refrigerator inside a standard variable-temperature insert cryostat. We modified the internal design of the MC for this experiment, as shown in Fig. 1c. Half of the MC was occupied by a displacer made of 6061 Al alloy with a 1.0 mm gap which was expected to be perpendicular to the incident neutron beam, although in our setup there was a slight misalignment with the gap deviating by 20° from the perpendicular plane. Additionally, there was a 3.5 mm diameter blind hole drilled 4 mm into the base of the MC in order to accommodate the still tube pickup. This is a distinctive feature in the neutron images, see for e.g., Fig. 2 where the helium mixture can be seen collecting in it. The base temperature of a standard KelvinoxJT^®^ is guaranteed below 25 mK with the specified 12.5 l of 28% ^3^He mixture. As a result of the MC modification, the measured base temperature increased to 37 mK and the ^3^He concentration had to be increased to 32% to account for the reduced mixing chamber volume.

### Image processing and analysis

For all neutron scans presented in this paper, to avoid overflows during subsequent processing, each captured scan first had outliers removed using a selective median filter. This determines if a pixel deviates by a grey value of at least + 100 (in the source 16-bit greyscale image from the camera) from the median grey value over a surrounding 5-pixel radius; and if it does, replaces it with the respective median value. Following this, all scans were calibrated by referencing them to the dark and open-beam images taken using the same pinhole and exposure time, such that pixel values of 0–1 (stored as a 32-bit float) are the neutron beam transmission coefficients. For scans taken in a time sequence using the same parameters, the variation in grey value over a nominally unchanging region was normalised from frame to frame to account for fluctuations in the neutron beam intensity. For presentation in this paper, all images exceeding a 600 dpi resolution at their displayed size have been binned (averaging of nearest neighbour pixels) by factor 2, and all results were rounded to 8-bit sample precision, stored as an integer (0–255) for compatibility reasons.

For the neutron image in Fig. 1b, 50 scans of the empty dilution refrigerator were taken, using D = 20 mm with an exposure time of 10 s, then the mean average of each pixel in this stack of 50 images was taken. The condensation images in Fig. 2 also use D = 20 mm and an exposure time of 10 s, with parts **d** and **e** being presented with adjusted contrast to highlight the partially filled mixture circuit. The corresponding mixture condensation video in Supplementary Video S1 was captured with an exposure time of 1 s to improve the time resolution. To achieve an acceptable file size for this ~ 1000 frame video (originally 16 GB), lossy H.264 compression was applied following binning by factor 2 and denoising using a non-local means spatial filter and a temporal low-pass filter.

For Fig. 3, 15 scans were captured after equilibration at each temperature using D = 20 mm and an exposure time of 30 s. Of these 15 scans, all those taken while the mixing chamber of the dilution refrigerator was within 2-standard deviations of the mean temperature had their pixels averaged and the result cropped to produce Fig. 3a–c. An offset was subtracted from these images to account for additional neutrons being scattered into the scintillator, resulting in a non-zero transmission being measured at the top left of the mixing chamber at base temperature (which would be expected to absorb all neutrons due to the 2 cm thickness of almost pure ^3^He). The overall attenuation of the neutron beam as it passed through the structure of the dilution refrigerator and helium mixture is given by the Beer–Lambert Law

2 $$\frac{I}{{I}_{0}}=\mathrm{exp}\left(-{\Sigma }_{s}{t}_{s}-{\Sigma }_{m}{t}_{m}\right)=\mathrm{exp}\left(-{\Sigma }_{s}{t}_{s}\right)\mathrm{exp}({-\Sigma }_{m}{t}_{m}),$$

where Σ_s,m_ are the macroscopic neutron absorption cross sections for the dilution refrigerator structure and mixture, respectively, and t_s,m_ are the thicknesses of the structure and mixture, respectively. By dividing the pixel values in the averaged images used in the figure, by those in the image of the empty refrigerator used for Fig. 1b, taking the natural logarithm and multiplying the result by − 1, the quantity Σ_m_t_m_ can be extracted. This quantity is plotted as a colour scale in Fig. 3e, in which the value 0 (corresponding to no additional absorption, i.e. where no mixture is present) was set to be transparent, allowing the colour-map of this quantity to be superimposed over the image of the empty dilution refrigerator in order to show the locations of these measurements more clearly. This quantity was averaged over the pixels contained within the black box in Fig. 3e for each of the temperature scans, giving the values shown in Fig. 3d.

The mixture macroscopic cross section is given by

3 $${\Sigma }_{m}={N}_{3}{\sigma }_{3}+{N}_{4}{\sigma }_{4},$$

where N_3,4_ are the atomic number densities of the ^3^He and ^4^He in the mixture, respectively. Also, σ_3_ and σ_4_ are the microscopic neutron absorption cross sections of ^3^He and ^4^He, respectively, scaled by the inverse of the neutron velocity from the data in^22^ for 1.798 Å neutrons for which σ_3_ = 5333 b and σ_4_ = 0.0075 b. Ignoring σ_4_ this therefore relates to the ^3^He concentration, c_3_, by

4 $${\Sigma }_{m}= \frac{{\rho }_{m}{\sigma }_{3}}{{m}_{3}}{c}_{3,}$$

where m_3_ is the atomic mass of ^3^He, and the overall helium mixture mass density is given by

5 $${\rho }_{m}={c}_{3}{\rho }_{3}+\left(1-{c}_{3}\right){\rho }_{4},$$

for ρ_3_ = 0.076 g/cm^3^ and ρ_4_ = 0.145 g/cm^3^, the T → 0 mass densities of ^3^He and ^4^He respectively, which vary little over the temperature range explored here^26^. In principle this should allow computation of c_3_ from the neutron data, however in this case it was found to produce unphysical values below the 6.6% theoretical minimum concentration^26^, likely due to the difficulties associated with estimating the neutron path length through the complex cross section of the dilution refrigerator, beam hardening^27^ and additional neutron scattering processes.

For Fig. 4, scans were captured using D = 40 mm and an exposure time of 10 s in order to achieve a reasonably low-noise image at a time resolution sufficient to resolve the single-shot process. To produce the images in the figure, and the corresponding Supplementary Video S2, a moving average over 10 successive scans was used in order to increase the visibility of the moving phase boundary against the noise. The images and Σ_m_t_m_ data for Fig. 5 was captured in an identical manner to that in Fig. 3.

## Supplementary Information

Supplementary Legends.

Supplementary Video S1.

Supplementary Video S2.

## References

[1] Pobell F (2007). Matter and Methods at Low Temperatures.

[2] Lounasmaa OV (1974). Experimental Principles and Methods Below 1 K.

[3] London, H. Comment. in *Proc. Int. Conf. on Low Temp. Phys.* (1951).

[4] London H, Clarke GR, Mendoza E (1962). Osmotic pressure of ^3^He in liquid ^4^He, with proposals for a refrigerator to work below 1 K. Phys. Rev..

[5] Das, P., Ouboter, R. B. de & Taconis, K. W. A realization of a London–Clarke–Mendoza type refrigerator. in *Proc. Low Temp. Phys. LT9* 1253–1255 (1965).

[6] Neganov B, Borisov N, Liburg M (1966). A method of producing very low temperatures by dissolving ^3^He in ^4^He. J. Exp. Theor. Phys..

[7] Hall HE, Ford PJ, Thompson K (1966). A helium-3 dilution refrigerator. Cryogenics.

[8] Wheatley JC, Rapp RE, Johnson RT (1971). Principles and methods of dilution refrigeration. II. J. Low Temp. Phys..

[9] Cousins DJ (1999). An advanced dilution refrigerator designed for the new Lancaster microkelvin facility. J. Low Temp. Phys..

[10] Uhlig K (2012). Cryogen-free dilution refrigerators. J. Phys..

[11] Koike Y (1999). A dilution refrigerator using the pulse tube and GM hybrid cryocooler for neutron scattering. Cryogenics.

[12] Castelvecchi D (2017). IBM’s quantum cloud computer goes commercial. Nature.

[13] Kandala A (2019). Error mitigation extends the computational reach of a noisy quantum processor. Nature.

[14] Batey G, Matthews AJ, Patton M (2014). A new ultra-low-temperature cryogen-free experimental platform. J. Phys. Conf. Ser..

[15] D’Addabbo A (2018). The CUORE cryostat. J. Low Temp. Phys..

[16] de Waard A (2006). Preparing for science run 1 of MiniGRAIL. Class. Quant. Gravity.

[17] Usenko O (2012). Development and Testing of the Gravitational Wave Antenna MiniGRAIL in Its Full Featured Configuration.

[18] Kirichek O (2019). Sample environment for neutron scattering experiments at ISIS. Neutron News.

[19] Frossati G, Godfrin H, Hebral B, Schumacher G, Thoulouze D (1977). Conventional cycle dilution refrigeration down to 2.0 mK. Phys. Ultralow Temps. Proc. Hakone Int. Symp..

[20] Frossati G (1978). Obtaining ultralow temperatures by dilution of ^3^He into ^4^He. J. Phys. Colloq..

[21] Minniti T, Watanabe K, Burca G, Pooley DE, Kockelmann W (2018). Characterization of the new neutron imaging and materials science facility IMAT. Nucl. Instrum. Methods Phys. Res. A.

[22] Sears VF (1992). Neutron scattering lengths and cross sections. Neutron News.

[23] Camus P (2014). Status of the closed-cycle dilution refrigerator development for space astrophysics. J. Low Temp. Phys..

[24] Hallock RB (1995). Chapter 5: The properties of multilayer ^3^He–^4^He mixture films. Prog. Low Temp. Phys..

[25] Alvesalo TA, Berglund PM, Islander ST, Pickett GR, Zimmermann W (1971). Specific heat of liquid ^3^He/^4^He mixtures near the junction of the λ and phase-separation curves I. Phys. Rev. A.

[26] Enss C, Hunklinger S (2005). Low-Temperature Physics.

[27] Bastürk M (2005). Analysis of neutron attenuation in boron-alloyed stainless steel with neutron radiography and JEN-3 gauge. J. Nucl. Mater..

