# Supplementary material for: Neutron imaging of an operational dilution refrigerator
Source: Sci Rep. 2022 Jan 21;12:1130. doi: 10.1038/s41598-022-05025-0 (PMC8783010; doi:10.1038/s41598-022-05025-0)
Supplement: Supplementary file 1 — Supplementary Legends. [file 41598_2022_5025_MOESM1_ESM.pdf]

Supplementary materials

Supplementary Video S1 (S1.mp4) is a video of the condensation process (50 MB, 86 s). The displayed parameters T_JT, T_still and T_MC are the temperatures of the Joule-Thompson mixture precooling stage, still and mixing chamber, respectively. The pressures P_cond, P_dump and P_still are for the mixture condenser, storage tank and still. The time is shown at the bottom left in the format HH:MM:SS.

Supplementary Video S2 (S2.mp4) is a video of the single-shot process (2.9 MB, 47 s). The displayed parameters T_MC and Mix 3He are the mixing chamber temperature and ^{3}He concentration, respectively. The time is also shown in the format HH:MM:SS.
